# Progress Toward Maternal and Neonatal Tetanus Elimination — Worldwide, 2000–2018

**DOI:** 10.15585/mmwr.mm6917a2

**Published:** 2020-05-01

**Authors:** Henry N. Njuguna, Nasir Yusuf, Azhar Abid Raza, Bilal Ahmed, Rania A. Tohme

**Affiliations:** ^1^Global Immunization Division, Center for Global Health, CDC; ^2^Immunization, Vaccines and Biologicals, World Health Organization, Geneva, Switzerland; ^3^Maternal, Newborn, and Adolescent Health Program Division, UNICEF, New York, New York.

Maternal and neonatal tetanus* (MNT) remains a major public health problem, with an 80%–100% case-fatality rate among neonates, especially in areas with poor immunization coverage and limited access to clean deliveries (i.e., delivery in a health facility or assisted by medically trained attendants in sanitary conditions) and umbilical cord care (*1*). In 1989, the World Health Assembly endorsed the elimination^†^ of neonatal tetanus (NT), and in 1999, the initiative was relaunched and renamed the MNT elimination^§^ initiative, targeting 59^¶^ priority countries (*1*). Elimination strategies include 1) achieving ≥80% coverage with ≥2 doses of tetanus toxoid-containing vaccine (TTCV) among women of reproductive age through routine immunization of pregnant women and supplementary immunization activities (SIAs)** in high-risk areas and districts^††^; 2) achieving care at ≥70% of deliveries by a skilled birth attendant (SBA)^§§^; and 3) enhancing surveillance for NT cases (*1*). This report summarizes progress toward achieving MNT elimination during 2000–2018. Coverage with ≥2 doses of TTCV (2 doses of tetanus toxoid [TT2+] or 2 doses of tetanus-diphtheria toxoid [Td2+]) among women of reproductive age increased by 16%, from 62% in 2000 to 72% in 2018. By December 2018, 52 (88%) of 59 priority countries had conducted TTCV SIAs, vaccinating 154 million (77%) of 201 million targeted women of reproductive age with TT2+/Td2+. Globally, the percentage of deliveries assisted by SBAs increased from 62% during 2000–2005 to 81% during 2013–2018, and estimated neonatal tetanus deaths decreased by 85%, from 170,829 in 2000 to 25,000 in 2018. By December 2018, 45 (76%) of 59 priority countries were validated by WHO as having achieved MNT elimination. To achieve elimination in the remaining 14 countries and sustain elimination in countries that have achieved it, implementation of MNT elimination strategies needs to be maintained and strengthened, and TTCV booster doses need to be included in country immunization schedules as recommended by the World Health Organization (WHO) (*2*). In addition, integration of maternal, newborn, and child health services with vaccination services is needed, as well as innovative approaches to target hard-to-reach areas for tetanus vaccination and community engagement to strengthen surveillance.

## Immunization Activities

To estimate TT2+/Td2+ vaccination coverage delivered through routine immunization services and the number of neonates protected at birth (PAB)^¶¶^ from neonatal tetanus, WHO and the United Nations Children’s Fund (UNICEF) use data from administrative records and vaccination coverage surveys reported annually by member countries (*3*). WHO and UNICEF also receive summaries of the number of women of reproductive age receiving TTCV during SIAs (*4*). During 2000–2018, coverage worldwide of women of reproductive age with TT2+/Td2+ increased by 16%, from 62% to 72% (*3*). In 2018, 17 (29%) of 59 priority countries achieved TT2+/Td2+ coverage ≥80%; in 39 of 48 (81%) priority countries where data were available,*** TT2+/Td2+ coverage increased compared with that in 2000. In 2018, the percentage of infants who were PAB was ≥80% in 46 (78%) of 59 priority countries (Table).

**TABLE:** Estimated coverage with ≥2 doses of tetanus toxoid-containing vaccine (TTCV) among women of reproductive age (WRA) administered through routine immunization services, estimated percentage of newborns protected at birth (PAB), number of WRA vaccinated with TTCV during supplementary immunization activities (SIAs), percentage of deliveries attended by a skilled birth attendant (SBA), and number of reported neonatal tetanus cases — 59 priority countries, 2000–2018

MNT elimination priority countries	WRA TT2+/Td2+ coverage (%)			Newborns PAB (%)			WRA vaccinated during TTCV SIAs*		SBA attendance at delivery (%)			No. of neonatal tetanus cases		
	Year		Change 2000–2018 (%)	Year		Change 2000–2018 (%)	No. of TT2+/Td2+ doses received	% vaccinated	Year^†^		Change 2000–2018 (%)	Year		Change 2000–2018 (%)
	2000	2018		2000	2018				2000	2018		2000	2018	
**Validated for MNT elimination by end-2018**														
Bangladesh	89	97	9	89	98	10	1,438,374	47	12	68	467	376	84	−78
Benin	81	69	−15	87	85	−2	1,399,461	97	66	78	18	52	13	−75
Burkina Faso	NA	92	NA	57	92	61	2,306,835	91	38	80	111	22	3	−86
Burma	81	89	10	79	90	14	8,170,763	87	57	60	5	41	22	−46
Burundi	28	90	221	51	90	76	679,222	55	25	85	240	16	0	−100
Cambodia	40	75	88	58	93	60	2,099,471	79	32	89	178	295	14	−95
Cameroon	40	66	65	54	85	57	2,687,461	85	56	65	16	279	27	−90
China	NA	NA	NA	NA	NA	NA	NA	NA	97	100	3	3230	83	−97
Comoros	40	78	95	57	85	49	160,767	55	62	NA	NA	NA	1	NA
Congo	39	83	113	67	85	27	273,003	91	83	91	10	2	0	−100
Côte d'Ivoire	78	85	9	76	85	12	5,924,527	85	63	74	17	30	17	−43
Egypt	71	NA	NA	80	86	7	2,518,802	87	61	92	51	321	2	−99
Equatorial Guinea	30	41	37	61	70	15	26,466	9	65	NA	NA	NA	6	NA
Eritrea	25	65	160	80	99	24	NA	NA	28	NA	NA	4	0	−100
Ethiopia	32	87	172	54	93	72	13,210,107	84	6	16	167	20	14	−30
Gabon	16	50	213	39	85	118	79,343	90	86	NA	NA	8	0	−100
Ghana	73	64	−12	69	89	29	1,666,666	87	47	78	66	80	9	−89
Guinea Bissau	NA	NA	NA	49	83	69	312,669	98	32	45	41	NA	0	NA
Haiti	NA	NA	NA	41	81	98	2,785,588	88	24	42	75	40	3	−93
India	80	81	1	85	90	6	7,643,440	94	43	81	88	3287	129	−96
Indonesia	81	47	−42	82	85	4	1,442,264	50	66	94	42	466	14	−97
Iraq	55	49	−11	75	75	0	111,721	96	65	96	48	37	3	−92
Kenya	51	61	20	68	88	29	4,463,695	67	42	62	48	1278	NA	NA
Laos	45	37	−18	58	90	55	968,323	90	17	64	276	21	16	−24
Liberia	25	74	196	51	89	75	288,984	57	51	61	20	152	14	−91
Madagascar	40	51	28	58	78	34	2,705,588	72	47	44	−6	13	30	131
Malawi	61	67	10	84	89	6	NA	NA	56	87	55	12	9	−25
Mauritania	NA	31	NA	44	80	82	586,277	76	53	69	30	NA	0	NA
Mozambique	61	85	39	75	86	15	605,640	79	48	73	52	42	160	281
Namibia	60	76	27	74	88	19	NA	NA	76	88	16	10	0	−100
Nepal	60	75	25	67	89	33	4,537,864	86	12	58	383	134	2	−99
Niger	31	94	203	63	81	29	2,184,277	92	16	40	150	55	9	−84
Philippines	58	48	−17	55	90	64	1,034,080	78	58	84	45	281	54	−81
Rwanda	NA	90	NA	81	95	17	NA	NA	31	91	194	5	2	−60
Senegal	45	65	44	62	95	53	359,845	92	58	68	17	0	6	NA
Sierra Leone	20	90	350	53	90	70	1,704,814	102	37	69	86	36	36	0
South Africa	65	NA	NA	68	90	32	NA	NA	91	97	7	11	0	−100
Tanzania	77	94	22	79	90	14	987,575	71	43	64	49	48	0	−100
Timor-Leste	NA	68	NA	NA	83	NA	24,141	53	18	57	217	NA	1	NA
Togo	47	76	62	63	83	32	262,130	87	35	45	29	33	14	−58
Turkey	36	55	53	50	95	90	1,242,674	58	83	98	18	26	0	−100
Uganda	42	66	57	70	85	21	2,448,527	86	39	74	90	470	78	−83
Vietnam	90	88	−2	86	94	9	367,842	69	59	94	59	142	37	−74
Zambia	61	76	25	78	85	9	330,030	81	42	63	50	130	71	−45
Zimbabwe	60	75	25	76	87	14	NA	NA	NA	78	NA	16	0	−100
**Not validated for MNT elimination by the end of 2018**														
Afghanistan	20	85	325	32	68	113	5,211,872	46	14	59	321	139	53	−62
Angola	NA	66	NA	60	78	30	7,097,552	84	NA	47	NA	131	86	−34
Central African Republic	20	89	345	36	60	67	804,984	78	32	NA	NA	37	39	5
Chad^§^	12	69	475	39	78	100	3,222,840	84	14	20	43	142	189	33
Democratic Republic of the Congo^§^	25	96	284	45	85	89	10,342,937	92	61	80	31	77	47	−39
Guinea	43	70	63	79	80	1	3,545,105	91	49	55	12	245	107	−56
Mali	62	60	−3	50	85	70	4,086,957	49	41	67	63	73	10	−86
Nigeria	NA	62	NA	57	60	5	4,986,353	84	34	43	26	1643	130	−92
Pakistan	51	60	18	71	85	20	21,143,148	87	23	69	200	1380	0	−100
Papua New Guinea	10	30	200	24	70	192	450,739	15	39	NA	NA	138	0	−100
Somalia	22	59	168	47	67	43	497,561	27	25	NA	NA	NA	NA	NA
South Sudan	NA	44	NA	NA	NA	NA	5,223,306	65	NA	NA	NA	NA	NA	NA
Sudan	34	51	50	NA	80	NA	4,780,345	89	NA	78	NA	88	NA	NA
Yemen	31	22	−29	54	70	30	3,043,456	52	27	45	67	174	116	−33
**All 59 priority countries**	**—**	**—**	**—**	—	—	—	**154,476,411**	—	—	—	—	**16,754**	**1,760**	**—**

**Abbreviations:** MNT = maternal and neonatal tetanus; NA = not available; Td2+ = 2 or more doses of tetanus and diphtheria toxoid-containing vaccine; TT2+ = 2 or more doses of TTCV.

* Includes first-year SIA conducted in Bangladesh in 1999 and first- and second-year SIAs conducted in Ethiopia in 1999.

^†^ Includes SBA attendance surveys conducted within 5 years for year 2000 and year 2018.

^§^ Validated for MNT elimination in 2019.

**FIGURE 1:**
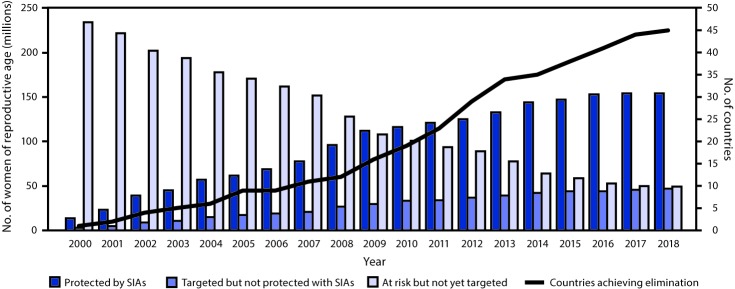
Number of women of reproductive age protected by TTCV* received during SIAs, number targeted but not yet vaccinated, number not yet targeted, and number of priority countries achieving maternal and neonatal tetanus elimination — worldwide, 2000–2018 **Abbreviations:** SIAs = supplementary immunization activities; TTCV = tetanus toxoid–containing vaccine. * 2 doses of tetanus toxoid (TT) or 2 doses of tetanus and diphtheria toxoids (Td).

By the end of 2018, 52 (88%) of 59 priority countries had conducted TTCV SIAs, and 154 million (77%) of the targeted 201 million women of reproductive age received at least 2 doses of TTCV (*4*). In 2018, 49 million women remain unreached by TTCV SIAs (Figure 1). Among the 52 countries that conducted TTCV SIAs, 29 (56%) vaccinated ≥80% of the targeted women with ≥2 doses of TTCV (Table). Among the 45 countries that achieved MNT elimination by the end of 2018, 38 (84%) had conducted TTCV SIAs. Among the seven countries that achieved elimination by the end of 2018 but did not conduct SIAs, six (China, Eritrea, Namibia, Rwanda, South Africa, and Zimbabwe) achieved MNT elimination through strengthening of routine immunization and reproductive health services; one country (Malawi) achieved elimination because women of reproductive age are targeted for vaccination during pregnancy, and 5 TTCV doses are provided in the routine vaccination schedule for children and adolescents.^†††^

## Surveillance Activities

**Reported NT cases and incidence.** WHO recommends nationwide case-based surveillance for NT, including zero-case reporting (submission of reports even if no NT cases are seen), active surveillance through regular site visits, and retrospective record review at major health facilities at least once a year (*2*). During 2000–2018, the number of reported NT cases worldwide (i.e., including nonpriority countries) decreased by 90% from 17,935 to 1,803 (*3*). In 2018, 13 (22%) of 59 priority countries reported zero NT cases (Table). The number of NT cases reported annually is likely to represent <11% of the actual number of NT cases occurring worldwide annually, because NT tends to occur in remote areas and cases might not be seen by health care workers (*5*).

**FIGURE 2:**
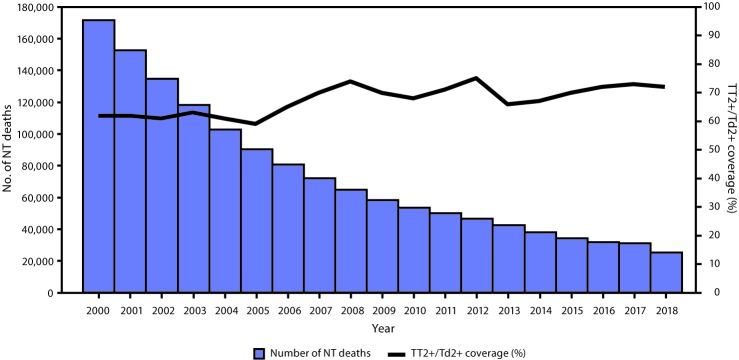
Estimated number of neonatal tetanus (NT) deaths and estimated coverage with ≥2 doses of tetanus toxoid (TT) or tetanus and diphtheria toxoids (Td)–containing vaccine (TT2+/Td2+) among women of reproductive age — worldwide, 2000–2018

**NT mortality estimates.** Because most NT deaths occur in the community and are not reported to WHO, NT deaths are usually estimated using mathematical models (*6*). During 2000–2018, the estimated number of NT deaths decreased by 85% from 170,829 to 25,000 (Figure 2). In 2018, neonatal tetanus accounted for 1% of major causes of neonatal deaths, a significant decrease compared with a 7% contribution to all-cause neonatal mortality in 2000.^§§§^

## Deliveries Assisted by Skilled Birth Attendants

WHO and UNICEF estimate the percentage of births attended by an SBA from health facility reports and coverage survey estimates shared by countries (*7*). During 2000–2018, the percentage of deliveries attended by an SBA increased by 31% from 62% during 2000–2005 to 81% during 2013–2018 (*7*). In 2018, among 51 priority countries with available data, ≥70% of deliveries were attended by an SBA in 24 (47%) countries (Table).

## Validation of Maternal and Neonatal Tetanus Elimination

WHO recommends the validation of MNT elimination when countries complete the implementation of planned elimination activities (*8*). The validation process involves a review of district-level core indicators, including reported NT cases per 1,000 live births, percentage of deliveries by SBA, TT2+/Td2+ coverage, and supplementary indicators, including TTCV SIA coverage, antenatal care coverage,^¶¶¶^ infant coverage with 3 doses of diphtheria-tetanus-pertussis vaccine, socioeconomic indices, urban versus rural status, field visits to assess the performance of the health system, validation surveys of districts with the most poorly performing MNT elimination indicators, and assessment of long-term plans for sustaining elimination (*9*). During 2000–2018, 45 (76%) of 59 priority countries were validated to have achieved MNT elimination, and 14**** remain to be validated (Table) (Figure 1). In addition, by 2018, three countries were validated to have achieved elimination in some regions: Pakistan (Punjab province), Mali (Southern regions), and Nigeria (South East zone).

## Discussion

There has been significant progress globally to eliminate MNT, and approximately 75% of the 59 priority countries were validated to have achieved MNT elimination by the end of 2018. The intensive targeting of “high-risk areas and districts” reached an estimated 154 million women of reproductive age with at least 2 doses of TTCV through SIAs, resulting in an 85% decline in the number of NT deaths annually during 2000–2018. Critical factors contributing to success include improvement in women’s access to education, country commitment to the implementation of recommended elimination strategies, timely availability of resources, good planning for SIAs, community engagement in elimination activities, strong monitoring and supervision of MNT elimination activities, and integrated delivery of antenatal care and tetanus vaccination services. Once countries are validated to have achieved MNT elimination, efforts to sustain elimination and broader tetanus control should continue, because tetanus cannot be eradicated from the environment.

MNT elimination validation assessments conducted in Cameroon and Timor-Leste, as well as Algeria and Djibouti (both validated before the 1999 relaunch of the initiative), showed that elimination was sustained; however, access to SBAs needed to be improved in Cameroon and Timor-Leste. Critical strategies for sustaining MNT elimination include strengthening routine immunization services for children and adolescents to receive a 3-dose primary TTCV series, and 3 TTCV booster doses at ages 12–23 months, 4–7 years, and 9–15 years to ensure long-term protection; antenatal screening of pregnant women for tetanus vaccination to ensure protection of neonates at birth; increased access to SBAs and clean delivery and cord care practices; strong tetanus surveillance; and periodic review of data to identify districts that are at risk for reemergence of MNT (*2*).

The findings in this report are subject to at least two limitations. First, TT2+/Td2+ coverage can underestimate true protection from tetanus, especially in countries with well-established vaccination programs, because it excludes women who were unvaccinated during pregnancy but were already protected through previous vaccination or had undocumented previous doses (*10*). Therefore, the percentage of PAB needs to be assessed, especially in countries that have achieved MNT elimination. Second, the number of neonatal tetanus cases and deaths are an underestimate of the actual number of NT cases because the majority of deaths occur in communities in areas underserved by the health care system (*5*).

Despite the progress made, the MNT elimination initiative still faces numerous challenges. Approximately 47 million women and their babies remain unprotected against tetanus, and 49 million women remain unreached by TTCV SIAs. Low TT2+/Td2+ coverage in these countries can be attributed to weak health systems, including conflict and security issues that limit access to vaccination services, competing priorities that limit the implementation of planned MNT elimination activities, and withdrawal of donor funding. Promoting institutional deliveries and ensuring the availability of clean delivery kits^††††^ for every home delivery would help MNT elimination and efforts to achieve the United Nations’ Sustainable Development Goal 3 to reduce maternal and neonatal mortality (https://www.un.org/sustainabledevelopment/health/). Innovative approaches to reach remote and unsafe areas could include the use of compact, prefilled autodisable devices; integration of reproductive, maternal, newborn, and child health services with vaccination services to optimize maternal immunization; and integration of TTCV SIAs with other SIAs, such as serogroup A meningococcal vaccine (MenA), measles-rubella, yellow fever, and polio campaigns. Efforts to strengthen NT surveillance through community engagement could serve as a platform for creating community-based surveillance systems for other diseases, and case-based surveillance for NT could be integrated with polio and measles case-based surveillance.^§§§§^

**Summary**

**What is already known about this topic?**

In 1999, the maternal and neonatal tetanus (MNT) elimination initiative was relaunched to focus on 59 priority countries that were still at risk for neonatal tetanus (NT).

**What is added by this report?**

During 2000–2018, 45 countries achieved MNT elimination, reported NT cases decreased 90%, and estimated deaths declined 85%. Despite this progress, some countries that achieved elimination are still struggling to sustain performance indicators; war and insecurity pose challenges in countries that have not achieved MNT elimination.

**What are the implications for public health practice?**

To maintain MNT elimination and to achieve it in remaining priority countries, sustained efforts are needed to enhance routine vaccination, embrace life-course vaccination, and develop innovative strategies for reaching underserved populations.

## Footnotes

* Maternal tetanus is defined as tetanus occurring during pregnancy or within 6 weeks of the end of pregnancy (birth, miscarriage, or abortion). Maternal tetanus infection occurs during abortion, miscarriages, or unhygienic delivery. Neonatal tetanus occurs during the first 28 days of life; neonatal tetanus infection occurs following cutting the umbilical cord under nonsterile conditions or applying nonsterile traditional remedies to the umbilical stump in an infant without passively (transplacentally) acquired maternal antibodies.

† Neonatal tetanus (NT) elimination is defined as the occurrence of less than one NT case per 1,000 live births per year in every district in every country.

§ NT elimination is considered a proxy for maternal tetanus elimination, and both share the same strategies for elimination.

¶ Initially, the total number of priority countries was 57. The creation of Timor-Leste in 2002 and South Sudan in 2011 increased the number of priority countries to 59.

** SIAs are mass vaccination campaigns that aim to administer doses of tetanus-containing vaccines to women of childbearing age.

†† High-risk areas and districts are defined as those in which the estimated NT case rate exceeds 1 per 1,000 live births, clean delivery coverage is less than 70%, and coverage with at least 3 tetanus toxoid-containing vaccine (TTCV) doses among pregnant women or women of reproductive age is less than 80% during the past 5 years.

§§ A skilled birth attendant is defined as a midwife, trained nurse, doctor, or a health extension or community health worker.

¶¶ Protected at birth (PAB) is defined as the status of an infant born to a mother who received 2 doses of tetanus toxoid or tetanus-diphtheria toxoid (TT/Td) during the last birth; 2 or more TT/Td doses, with the last dose received ≤3 years before the last delivery; 3 or more doses with the last dose received ≤5 years earlier; 4 or more doses with the last dose received ≤10 years earlier; or receipt of 5 or more previous doses.

*** Angola, Burkina Faso, China, Egypt, Guinea Bissau, Haiti, Mauritania, Nigeria, Rwanda, Timor-Leste, South Africa, and South Sudan had missing TT2+/Td2+ coverage data for the year 2000 or 2018.

††† https://cdn1.sph.harvard.edu/wp-content/uploads/sites/2413/2015/12/Nina-Schwalbe-1.pdf; https://apps.who.int/iris/bitstream/handle/10665/232360/WER7901_02_2-6.PDF?sequence=1&isAllowed=y.

§§§ https://www.unicef.org/media/60561/file/UN-IGME-child-mortality-report-2019.pdf.

¶¶¶ Antenatal care coverage is the percentage of women aged 15–49 years with a live birth who had received antenatal care provided by skilled health personnel (doctor, nurse, or midwife) at least once during the pregnancy.

**** Chad and the Democratic Republic of the Congo were validated in 2019, leaving 12 countries not validated by December 2019.

†††† https://www.unfpa.org/sites/default/files/resource-pdf/RH%20kits%20manual_EN_0.pdf.

§§§§ https://www.who.int/immunization/monitoring_surveillance/burden/vpd/WHO_SurveillanceVaccinePreventable_14_NeonatalTetanus_R1.pdf.
